# Brain‐Penetrating and Disease Site‐Targeting Manganese Dioxide‐Polymer‐Lipid Hybrid Nanoparticles Remodel Microenvironment of Alzheimer's Disease by Regulating Multiple Pathological Pathways

**DOI:** 10.1002/advs.202207238

**Published:** 2023-02-19

**Authors:** Elliya Park, Lily Yi Li, Chunsheng He, Azhar Z. Abbasi, Taksim Ahmed, Warren D. Foltz, Regan O'Flaherty, Maham Zain, Robert P. Bonin, Andrew M. Rauth, Paul E. Fraser, Jeffrey T. Henderson, Xiao Yu Wu

**Affiliations:** ^1^ Leslie Dan Faculty of Pharmacy University of Toronto 144 College St Toronto ON M5S 3M2 Canada; ^2^ Department of Radiation Oncology University Health Network 149 College St Toronto ON M5T 1P5 Canada; ^3^ Tanz Centre for Research in Neurodegenerative Diseases Department of Medical Biophysics University of Toronto 135 Nassau St Toronto ON M5T 1M8 Canada; ^4^ Departments of Medical Biophysics and Radiation Oncology University of Toronto 101 College St Toronto ON M5G 1L7 Canada

**Keywords:** Alzheimer's disease, brain microenvironment, nanomedicine, neuroinflammation, oxidative stress, vascular function

## Abstract

Finding effective disease‐modifying treatment for Alzheimer's disease remains challenging due to an array of factors contributing to the loss of neural function. The current study demonstrates a new strategy, using multitargeted bioactive nanoparticles to modify the brain microenvironment to achieve therapeutic benefits in a well‐characterized mouse model of Alzheimer's disease. The application of brain‐penetrating manganese dioxide nanoparticles significantly reduces hypoxia, neuroinflammation, and oxidative stress; ultimately reducing levels of amyloid *β* plaques within the neocortex. Analyses of molecular biomarkers and magnetic resonance imaging‐based functional studies indicate that these effects improve microvessel integrity, cerebral blood flow, and cerebral lymphatic clearance of amyloid *β*. These changes collectively shift the brain microenvironment toward conditions more favorable to continued neural function as demonstrated by improved cognitive function following treatment. Such multimodal disease‐modifying treatment may bridge critical gaps in the therapeutic treatment of neurodegenerative disease.

## Graphical abstract

Multifunctional brain‐penetrating bioactive manganese dioxide nanoparticles modify brain microenvironment of Alzheimer's disease (AD) by reducing inflammation and oxidative stress and improving vascular blood flow and lymphatic clearance of amyloid. This multitargeted strategy shifts the brain microenvironment toward more favorable conditions for neural function and consequent memory improvement. This study may bridge critical gaps in finding disease‐modifying treatment for neurodegenerative disease.

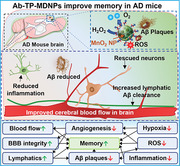

## 1 Introduction

**Figure 1:**
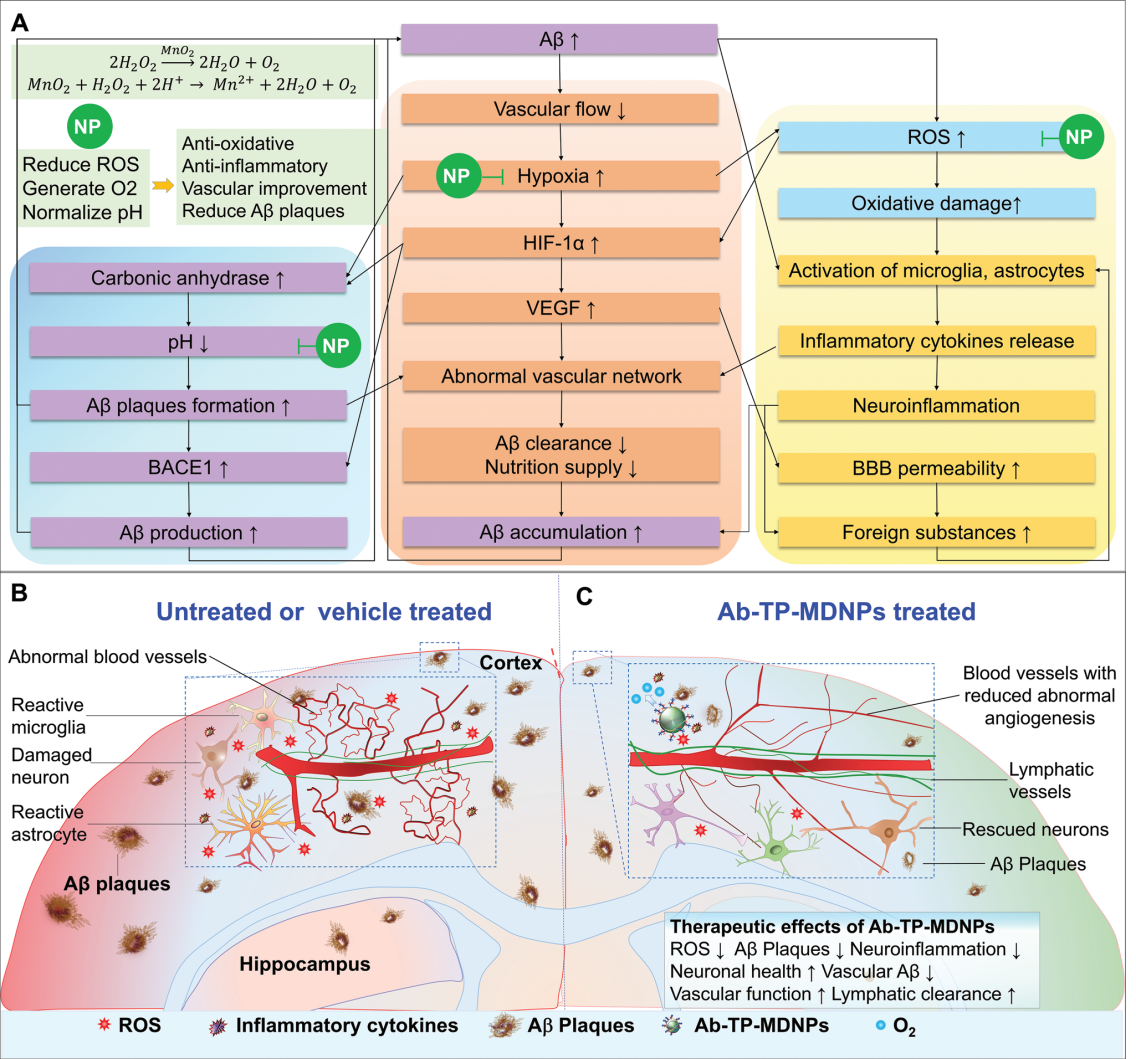
Mechanisms of multifunctional effects of Ab‐TP‐MDNPs (NP) on remodeling the AD brain microenvironment. A) Diagram of feedback loop for multiple pathological pathways of hypoxia (middle panel, orange) and oxidative stress (right panel, blue) that promote abnormal angiogenesis (middle panel, orange), A*β* generation and accumulation (left panel, purple), neuroinflammation (lower right panel, yellow), and oxidative stress (upper right panel, blue) which can be intercepted by the NP on multiple fronts simultaneously by quenching ROS (H_2_O_2_), generating O_2_, and normalizing pH (upper left panel, green). Brain hypoxia caused by A*β* accumulation and abnormal vasculature is attenuated via NP reoxygenation and this, together with ROS reduction, decreases HIF‐1*α* and its actions on BACE1, carbonic anhydrase (CA9), and VEGF, ultimately resulting in less A*β* generation, more A*β* clearance, and lowered oxidative stress and neuroinflammation. B) Schematic illustration of untreated or vehicle‐treated brain microenvironment in AD with denser and larger A*β* plaques, higher oxidative stress and neuro‐inflammation, and abnormal vasculature; and C) Ab‐TP‐MDNPs treated brain with fewer A*β* plaques, normalized vascular structure, lower oxidative stress, and neuroinflammation.

Alzheimer's disease (AD) is the most common form of dementia, principally manifested by progressive memory loss and cognitive impairments, affecting more than 50 million people worldwide.^[^ ^1^ ^]^ Clinically AD is characterized by the asymmetric deposition of neurotoxic amyloid *β* (A*β*) and hyper‐phosphorylated tau accumulations in the brain.^[^ ^2^ ^]^ Numerous attempts have been made to reduce levels of these neurotoxic proteins with minimal success, perhaps due to reductions of these proteins alone does not eliminate the root causes of AD, relating to pathological oxidative stress and neuroinflammation.^[^ ^3^, ^4^ ^]^ Mounting evidence suggests that a key element in the resulting neuropathology is connected to the induction of oxidative stress, neuroinflammation, and hypoxia which appear to exist chronically in the brains of AD patients; beginning well before pathologic symptoms emerge.^[^ ^5^, ^6^ ^]^ Such stressors can initiate and amplify numerous signaling pathways promoting progression of AD (**Figure** **1**). Additionally, age‐related vascular impairment due to plaque deposition and other causes can promote localized hypoxia, further exacerbating neuronal stress. Reduction of oxygen concentrations to less than 6% (≈40 mm Hg) results in an exponential increase in hypoxia‐inducible factor‐1*α* (HIF‐1*α*) as well as elevation of the angiogenic cytokine, vascular endothelial growth factor (VEGF).^[^ ^7^ ^]^ Reactive oxygen species (ROS) further upregulates HIF‐1*α*, neuro‐inflammation and A*β* generation via activation of beta‐secretase 1 (BACE1).^[^ ^8^, ^9^ ^]^ Pathologic upregulation of VEGF accelerates angiogenesis, leading to the formation of abnormal microvasculature, further interfering with blood flow through plaque‐bound vessels.^[^ ^10^, ^11^ ^]^ Such vascular dysfunction plays a significant role in promoting cognitive impairment in AD and limits A*β* clearance, further elevating hypoxia and oxidative stress.^[^ ^12^, ^13^, ^14^ ^]^ A similar dysfunction is also observed in the cerebral lymphatics of AD patients, a major A*β* clearance route carrying cerebrospinal fluid (CSF) and associated proteins from the brain.^[^ ^15^, ^16^ ^]^ Consequently, impaired A*β* clearance in this system further advances oxidative stress, neuroinflammation, and vascular dysfunction.^[^ ^17^ ^]^ These phenomena represent part of positive feedback loop leading to neural dysfunction in AD. Various treatments against the above stressors have been investigated but have led to minimal benefit, attributable to the multifactorial nature of this neurological disorder.^[^ ^3^, ^18^, ^19^ ^]^

In this respect, many prior studies have targeted dominant elements of AD pathology such as A*β* production and deposition, tau aggregation, angiogenesis, neuroinflammation, or neurotransmitter deficit.^[^ ^20^ ^]^ These approaches alone provide insufficient clinical benefits due to the complex inter‐relationships between pathological pathways. Therefore, recent studies have explored combinatorial therapies targeting more than one pathway.^[^ ^21^ ^]^ However, such combinatorial therapies still exhibit issues addressing complex multifaceted pathogenic processes due to target limitations. Additionally, the blood–brain barrier (BBB) further restricts the availability of therapeutics for treating this disorder.^[^ ^22^ ^]^

In the current study, we propose a new multifunctional strategy aimed at inhibiting several key pathologic signaling mechanisms observed in AD using manganese dioxide nanoparticles (MDNPs).^[^ ^23^ ^]^ These nanoparticles (NP) are functionalized with anti‐A*β* antibody (4G8, designated Ab) and are composed of a BBB‐crossing terpolymer (TP)‐MDNPs (Ab‐TP‐MDNPs). These Ab‐TP‐MDNPs effectively cross the BBB^[^ ^24^ ^]^ reaching disease‐affected areas of the brain and binding to A*β* plaques or oligomers to react with local ROS to generate oxygen (O_2_).^[^ ^23^ ^]^ As a result, Ab‐TP‐MDNPs not only act as a potent antioxidant (quenching locally generated H_2_O_2_), but also act as an O_2_ generator, influencing multiple AD pathologies such as those associated with low oxygen (hypoxia) and elevated ROS (Figure 1). Using both molecular and functional studies we demonstrate that treatment with intravenously (IV) injected Ab‐TP‐MDNPs reduces hypoxia, BACE1, VEGF, proinflammatory cytokines, vascular, and lymphatic dysfunction in a transgenic mouse model of AD (TgCRND8). Importantly this treatment also increases lymphatic clearance of A*β* through CSF drainage, thereby reducing amounts of A*β* plaques and vascular A*β* in the brain. As a result, Ab‐TP‐MDNPs improved cognitive function in treated animals. Ab‐TP‐MDNPs thus show potential as a multifunctional therapeutic strategy for treatment of AD.

## 2 Results

### 2.1 Characterizations of Ab‐TP‐MDNPs

The nanoparticles with (Ab‐TP‐MDNPs) or without (TP‐MDNPs) antibody functionalization were prepared based on the previously described method(23). A spherical and monodispersed nanoparticles were formed with particle size of 118 and 117 nm, and polydispersity indices (PDI) of 0.25 and 0.26 for Ab‐TP‐MDNPs and TP‐MDNPs, respectively, measured by dynamic light scattering (DLS) (Figure S1A, Supporting Information). DLS results align with the transmission electron micrographs images representing uniform and spherical shapes (Figure S1B, Supporting Information). Additionally, the zeta potential of Ab‐TP‐MDNPs and TP‐MDNPs were −40 and −42 mV, respectively, indicative of stable nanoparticle formation. To improve the stability of nanoparticle, the final suspension is lyophilization using a freeze‐drying technique. Lyophilization preserved the characterization of the nanoparticles as no significant change in particle size was observed following this process while increasing availability for the enhanced clinical use. CNS penetrating ability of Ab‐TP‐MDNPs was verified in order to confirm our previous findings. Both Ab‐TP‐MDNPs and TP‐MDNPs were subsequently detected within the brain (Figure S1C, Supporting Information). Antibody functionalized NPs demonstrated increased bioavailability of nanoparticles in the CNS. Ab‐TP‐MDNPs showed a 1.51‐fold increased absorption in the brain compared to TP‐MDNPs 15 min postadministration (Figure S1D,E, Supporting Information). Antibody functionalization also enhanced retention in brain, with 2.92‐fold brain fluorescence signal when compared to TP‐MDNPs post IV administration (Figure S1D,E, Supporting Information). Confocal laser scanning microscopy (CLSM) of brain tissue slices demonstrated that antibody functionalization enables the nanoparticles to colocalize with A*β* plaques (Figure S1F, Supporting Information).

### 2.2 Ab‐TP‐MDNPs Reduce Hypoxia and Oxidative Stress

**Figure 2:**
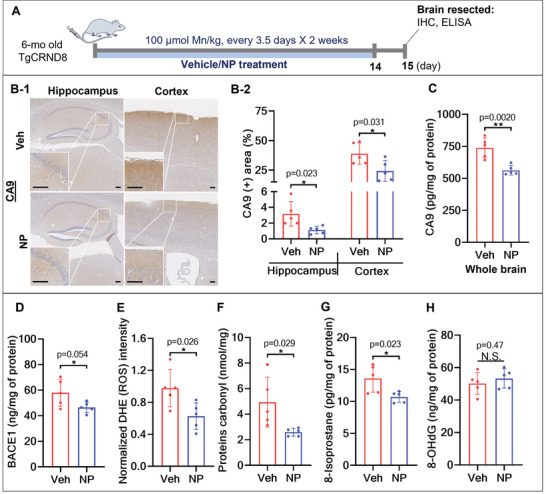
Ab‐TP‐MDNPs reduce hypoxia and oxidative stress. A) Six‐month (mo) old AD mice (random distribution, male and female mice) were treated IV with 100 µmol Mn kg^−1^ body weight (b.w.) of Ab‐TP‐MDNPs (NP) or vehicle (Veh) (4% dextrose) (control group) twice weekly for 2 weeks and measurements were made on day 15. B) Representative photos B‐1) and quantification B‐2) by immunohistochemistry of CA9 (hypoxia marker), in the hippocampus and cortex of AD mouse brain. Scale bars equal 100 µm in the two magnifications shown. C) Levels of CA9 as measured by ELISA in AD brain homogenates. D) Levels of BACE1 as measured by ELISA in AD brain homogenates. E) ROS levels indicated by dihydroethidium (DHE) fluorescence intensity in AD brain homogenates. F–H) Levels of cellular oxidative markers in AD brain homogenates. F) Protein oxidation levels indicated by protein carbonylation, G) lipid oxidation levels indicated by 8‐isoprostane measured by ELISA, and H) DNA oxidation levels indicated by 8‐OHdG measured by ELISA. The data are presented as mean ± SD (*n* = 5 per group). Individual values are shown (dots) for vehicle and NP‐treated mice. Asterisk(s) (*) denotes a significant difference at *p < 0.05, **p < 0.01) compared to vehicle treatment. N.S.—not significant.

We first investigated the direct effect(s) of Ab‐TP‐MDNPs on reducing hypoxia and oxidative stress in the AD brain in a mouse model of AD (TgCRND8).^[^ ^23^ ^]^ Therapeutic effects were evaluated following IV injection of Ab‐TP‐MDNPs or vehicle (4% dextrose) into TgCRND8 mice and wild type littermates (WT) twice a week (every 3.5 days) for 2 weeks (**Figure** **2**A). Duration, frequency, and dosage of treatment were chosen based on previously determined pharmacokinetic and toxicity profiles of Ab‐TP‐MDNPs and the biomarkers studied (unpublished data and Figure S1, Supporting Information). After 2 weeks of treatment with Ab‐TP‐MDNPs, levels of carbonic anhydrase 9 (CA9), a hypoxic marker, were reduced within the cortex by 38%, and the hippocampus by 65% (Figure 2B); consistent with Ab‐TP‐MDNPs‐targeted primary regions of AD pathology. We observed strong CA9 expression in the hippocampal CA1 (cornu ammonis 1) region, which is particularly vulnerable to hypoxic damage,^[^ ^25^ ^]^ was resolved by Ab‐TP‐MDNPs treatment (Figure 2B). Enzyme‐linked immunoassay (ELISA) of whole brain homogenates demonstrated levels of CA9 were reduced 23% after Ab‐TP‐MDNPs treatment (Figure 2C). BACE1 activity in the brain, a major contributor to A*β* generation, was also reduced by Ab‐TP‐MDNPs treatment by 20% (Figure 2D). Consistent with this, total ROS levels in the brain were diminished by Ab‐TP‐MDNPs treatment by 36% (Figure 2E). The level of HIF‐1*α*, a biomarker of hypoxia, showed a decreasing trend in brain following Ab‐TP‐MDNPs treatment with statistical insignificance (Figure S3A, Supporting Information). Analysis of oxidative stress markers indicated that Ab‐TP‐MDNPs treatment attenuated oxidative damage of proteins (protein carbonylation) by 47% and lipids (8‐isoprostane) by 21%, but did not appear to alter DNA (8‐Hydroxy‐2'‐deoxyguanosine (8‐OHdG) levels (Figure 2F–H). These results demonstrate that multiple IV injections of Ab‐TP‐MDNPs can effectively reduce oxidative stress and hypoxia in the AD brain. Furthermore, Ab‐TP‐MDNPs treatment in murine primary cortical neurons demonstrated a protective effect on dendrites following H_2_O_2_ neurotoxic challenge (Figure S3B, Supporting Information).

### 2.3 Ab‐TP‐MDNPs Reduces Neuroinflammation

**Figure 3:**
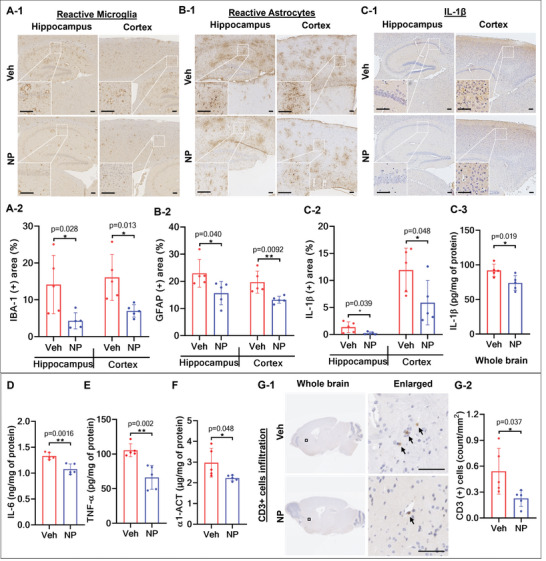
Ab‐TP‐MDNPs reduce neuroinflammation. Six‐month‐old AD mice were treated IV with 100 µmol Mn kg^−1^ b.w. of Ab‐TP‐MDNPs or Veh control twice weekly for 2 weeks (Treatment schedule follows that in Figure 2A). A) Representative photos A‐1) and quantification A‐2) of IHC staining of reactive microglia (ionized calcium binding adaptor molecule 1 (IBA‐1) in hippocampus and cortex of AD mouse brain. Scale bars equal 100 µm. B) Representative photos B‐1) and quantification B‐2) of IHC of reactive astrocytes (glial fibrillary acidic protein (GFAP)) in the hippocampus and cortex of AD mouse brain. Scale bars equal 100 µm. C) Representative photos C‐1) and quantification C‐2) of IHC of IL‐1*β* in the hippocampus and cortex of AD mouse brain. Scale bars equal 100 µm. IL‐1*β* level in AD brain homogenates measured by ELISA C‐3). D–F) Levels of pro‐inflammatory cytokines (D: IL‐6, E: TNF‐*α*, and F: *α*1‐ACT) measured by ELISA in the AD brain homogenates. G) Representative photos G‐1) and quantification G‐2) of infiltrated CD3^+^ cells in the AD mouse brain. Scale bars equal 50 µm. Arrows indicate CD3^+^ cells in the brains. Data are presented as mean ± SD (*n* = 5 per group). Individual values are shown (dots) for vehicle and NP treated mice. Asterisk(s) (*) denotes a significant difference at *p < 0.05, **p < 0.01) compared to vehicle treatment. N.S.—not significant.

The observed reductions in oxidative stress and hypoxia markers in Ab‐TP‐MDNPs treated mice prompted us to study additional interactions known to be altered by ROS and hypoxia. The results showed that Ab‐TP‐MDNPs treatment attenuated neuroinflammation in the AD brain, with 70% and 57% reductions of reactive microglia in the hippocampus and cortex, respectively (**Figure** **3**A‐2). Similarly, reductions of reactive astrocytes in the hippocampus and cortex by 32% and 33% respectively (Figure 3B‐2). We then measured neuroinflammatory cytokines related to AD progression. Ab‐TP‐MDNPs treatment reduced IL‐1*β* in the hippocampus and the cortex by 83% and 51%, respectively (Figure 3C‐2) consistent with observed reductions in activated microglia and astrocytes. A significant decrease in IL‐1*β* (20%, Figure 3C‐3), IL‐6 (19%, Figure 3D), tumor necrosis factor‐*α* (TNF‐*α*, 37%, Figure 3E), and *α*1‐antichymotrypsin (*α*1‐ACT: 25%, Figure 3F) in AD brains was also observed following treatment with Ab‐TP‐MDNPs as measured by ELISA in whole brain homogenates. Infiltration of cluster of differentiation 3 (CD3^+^) cells into the brain was also reduced (58% in the whole brain) in Ab‐TP‐MDNPs treated AD brain (Figure 3G) suggesting an anti‐inflammatory effect. Overall, these results demonstrate that Ab‐TP‐MDNPs can significantly reduce inflammatory cytokines and neuroinflammation in the AD brain.

### 2.4 Ab‐TP‐MDNPs Improved Vascular Flow and BBB Integrity

**Figure 4:**
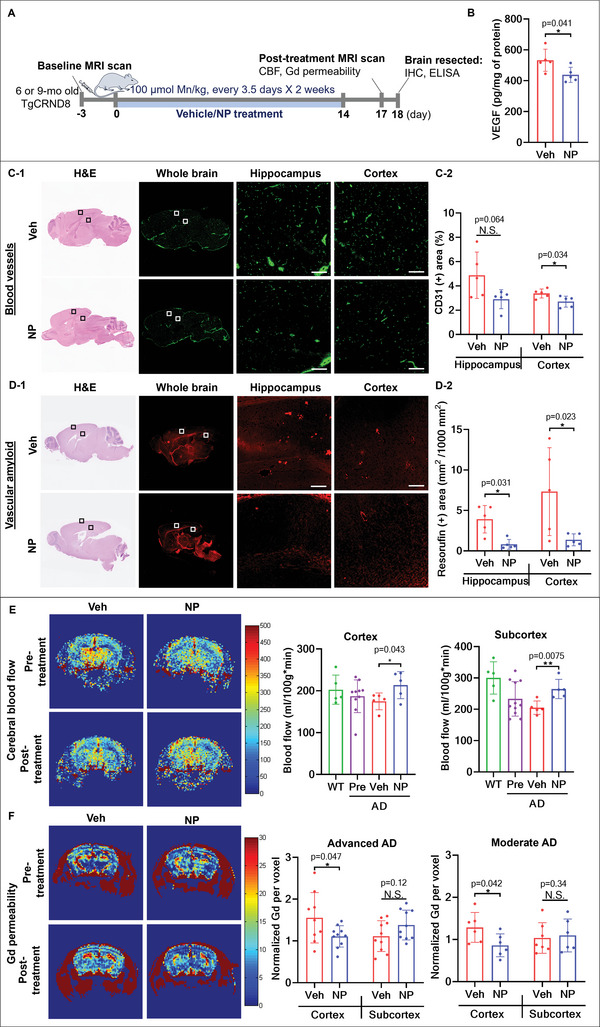
Ab‐TP‐MDNPs normalize vasculature and improve vascular function. A) Six or 9‐mo‐old AD mice were treated IV with 100 µmol Mn kg^−1^ b.w. of Ab‐TP‐MDNPs or Veh control twice weekly for 2 weeks and measurements were taken before and after the treatment. B) Levels of VEGF measured by ELISA in AD brain homogenates. C) Hematoxylin/eosin overview highlighting enlargement of areas stained for CD31 C‐1), to assess microvessels in the hippocampus and cortex of AD mouse brains. Scale bars equal 100 µm results are quantified in (C‐2). D) Hematoxylin/eosin overview with enlargements demonstrating resorufin staining for cerebrovascular A*β* in the hippocampus and cortex of AD mouse brains D‐1). Scale bars equal 100 µm, results quantified in (D‐2). E) Cerebral blood flow in the cortex and subcortex as measured by MR imaging using FAIR‐FISP in advanced (9‐mo) AD mouse brains pre‐ and post‐treatment with either Ab‐TP‐MDNPs (NP) or Veh and WT mouse brains without treatment. F) BBB permeability of cortex and subcortex measured by R1 difference maps (% change) following Gd‐DTPA injection in moderate (6‐mo) and advanced (9‐mo) AD mice, reflecting changes in Gd‐DTPA extravasation into the brain parenchyma. Data are presented as mean ± SD (*n* = 5 per group for A–D and 5–10 per group for E,F). Individual values are shown (dots) for vehicle and NP treated mice. Asterisk(s) (*) denotes a significant difference at *p < 0.05, **p < 0.01) compared to vehicle treatment. N.S.—not significant.

Hypoxia and ROS play key roles in the development of vascular dysfunction by disrupting vascular reactivity and vascular structure via VEGF activation. Also, ROS and neuroinflammation damage the neurovascular unit. Therefore, we sought to explore the effect of Ab‐TP‐MDNPs treatment in reversing vascular dysfunction, which is frequently observed in the brains of AD patients.^[^ ^14^, ^26^ ^]^ First, we hypothesized that a significant reduction in hypoxia and ROS following Ab‐TP‐MDNPs treatment would help normalize VEGF expression in the brains of AD mice. Following IV injection of Ab‐TP‐MDNPs twice weekly for 2 weeks (**Figure** **4**A), the expression of VEGF observed in AD mouse brains was reduced by 18% compared to the vehicle‐treated group (Figure 4B). VEGF is a strong angiogenic cytokine and its prolonged activation enhances the growth of immature vasculature, which has been observed in AD brains.^[^ ^13^, ^27^ ^]^ We therefore examined the density of microvasculature via CD31 staining in AD brains (Figure 4C). Consistent with VEGF expression results, Ab‐TP‐MDNPs treatment decreased the density of microvessels in the brain cortex area by 20%. In addition, a significant load of cerebrovascular A*β* plaques were observed in the vehicle‐treated AD brains, whereas in the Ab‐TP‐MDNPs treatment group the levels were 41% and 46% lower in the hippocampus in the cortex, respectively (Figure 4D).

Finally, we extended our investigation to examine functional effects of Ab‐TP‐MDNPs treatment on CBF and BBB permeability via magnetic resonance imaging (MRI) using a 7 Tesla micro‐MRI system (see schedule Figure 4A). As shown in Figure 4E, impaired blood flow within the advanced (9‐mo old) AD mouse brains was significantly improved in both cortical (18%) and subcortical regions (23%) following Ab‐TP‐MDNPs treatment. The observed increase in blood flow is attributable to the effect of Ab‐TP‐MDNPs treatment on normalizing the vascular structure. Permeability of the BBB, which is known to increase with AD progression, was evaluated at both moderate (6‐mo old) and advanced (9‐mo old) TgCRNBD8 AD stages following high‐dose injection of gadolinium‐diethylenetriamine penta‐acetate (Gd‐DTPA).^[^ ^13^ ^]^ Compared to vehicle controls, R1 elevations in the cortex, reflecting increased Gd‐DTPA extravasation into the brain parenchyma, were reduced as a result of Ab‐TP‐MDNPs treatment at both stages (advanced: 29%, moderate: 33%) (Figure 4F). The improvement in BBB integrity could also protect the brain from peripheral injury and infiltrators that promote inflammation and might be related to decreased CD3^+^ cell infiltration (Figure 3G).

### 2.5 Ab‐TP‐MDNPs Treatment Increases Lymphatic Clearance of A*β*

**Figure 5:**
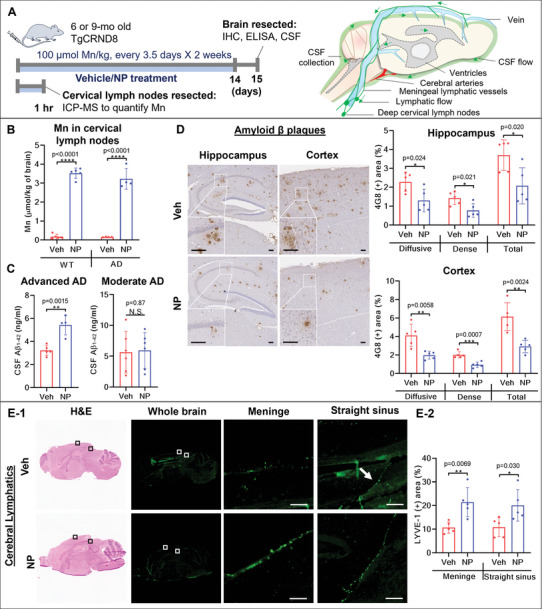
Ab‐TP‐MDNPs increases lymphatic A*β* clearance. A) (Left top) Six or 9‐mo old AD mice were treated IV with 100 µmol Mn kg^−1^ b.w. of Ab‐TP‐MDNPs or Veh control twice weekly for 2 weeks and CSF and brain were harvested. (Left bottom) Six‐mo old AD or WT mice received single IV administration of 500 µmol Mn kg^−1^ b.w of Ab‐TP‐MDNPs, cervical lymph nodes were collected 60 min after administration for B. (Right top) Diagram of the brain and its lymphatic flow and area of CSF collection. B) Biodistribution of Ab‐TP‐MDNPs in cerebral lymphatics in AD and WT mice. C) The levels of A*β*_1‐42_ in CSF in advanced (9‐mo) and moderate (6‐mo) AD mice after Veh or Ab‐TP‐MDNPs treatment. D) Immunohistochemistry of 4G8 measuring A*β* in the hippocampus and the cortex of moderate stage of AD mouse brains. Scale bars equal 100 µm. E) Hematoxylin/eosin overview with enlargements demonstrating LYVE‐1 staining for cerebral lymphatics in the meninge and straight sinus of AD mouse brains E‐1). Scale bars equal 100 µm, results quantified in (E‐2). The data are presented as mean ± SD (*n* = 4–5 per group). Individual values are shown (dots) for Veh and NP treated mice. The asterisk (*) denotes significant difference at *p < 0.05, **p < 0.01, ***p < 0.001, ****p < 0.0001) as compared to vehicle treatment. N.S.—not significant.

The ventricular lymphatic pathway within the brain is another significant route of A*β* and tau clearance and it is known to be compromised as AD progresses.^[^ ^15^, ^16^ ^]^ The clearance of CSF through these perivascular spaces is driven via arterial pulsatility.^[^ ^28^ ^]^ We have previously demonstrated high selectivity and reactivity of Ab‐TP‐MDNPs in the CSF of AD brains via MRI.^[^ ^23^ ^]^ Given the effects on vascular perfusion and its selectivity in the CSF, we examined whether treatment with Ab‐TP‐MDNPs could assist in normalizing lymphatic function. To test this hypothesis, we first validated the existence of Ab‐TP‐MDNPs in the brain lymphatics using 6‐mo old AD and WT mice (**Figure** **5**). One hour after Ab‐TP‐MDNPs administration, cervical lymph nodes which drains brain lymphatics (Figure 5A, right), were collected and Mn levels were measured using inductively coupled plasma atomic emission spectroscopy (ICP‐AES). The levels of Mn from Ab‐TP‐MDNPs were calculated by deducting Mn levels from that of vehicle‐treated littermates. These data demonstrate that Mn in Ab‐TP‐MDNPs can enter the lymphatic system of the brain of both AD and WT mice (Figure 5B). Since lymphatic vessels convey CSF, we measured A*β* clearance via CSF in both advanced (9‐mo) and moderate (6‐mo) stages of AD (Figure 5C). Interestingly, Ab‐TP‐MDNPs treatment resulted in significantly greater CSF A*β* clearance in advanced AD cases (by 69%) compared to vehicle, but less in moderate AD mice. This result may be attributable to the fact that, in advanced AD, higher levels of targetable A*β* are present within tissues, allowing greater opportunity for the uptake of A*β* by Ab‐TP‐MDNPs. Based on the effects of Ab‐TP‐MDNPs in improving CSF A*β* clearance as well as vascular perfusion shown above, we sought to evaluate the effects of Ab‐TP‐MDNPs in reducing A*β* plaques in AD brains. The data indicated that Ab‐TP‐MDNPs treatment reduced the amount of diffuse and dense A*β* plaques by 54% and by 52%, respectively, and total plaques by 53% in the cortex and 44% in the hippocampus in AD mice at moderate stage (≈6‐mo age) (Figure 5D). In advanced AD mice (9‐mo), Ab‐TP‐MDNPs treatment showed a trend toward reducing both types of A*β* plaques, however the differences observed were statistically insignificant (Figure S4B, Supporting Information).

We further investigated lymphatics in the AD brain by staining for lymphatic vessel endothelial hyaluronan receptor‐1 (LYVE‐1) antibody, analyzing lymphatics proximal to blood vessels in 6‐mo AD mice after the treatment (Figure 5E). The data demonstrate that Ab‐TP‐MDNPs treatment significantly improved lymphatic structure as evidenced by increased morphological uniformity and lymph vessel density of LYVE‐1^+^ lymphatic vessels in the AD brain by 100% in the inner meningeal layer and by 86% in the straight sinus as compared to vehicle treatment. These results suggest that normalization of the lymphatic structure by Ab‐TP‐MDNPs treatment could improve lymphatic clearance of soluble A*β*. Further analysis of lymphatics proximal to blood vessels in advanced AD (9‐mo AD mice) demonstrated greater deterioration in lymphatic vessels with 2–4% LYVE‐1^+^ area (Figure S4A, Supporting Information) compared to ≥10% in 6‐mo AD mice (Figure 5E‐2). These findings align with more impaired CSF A*β* clearance as AD progresses (Figure 5C). However, the effects of Ab‐TP‐MDNPs treatment, though showed a positive trend toward improvement in this population, was less significant than that at moderate AD stages, which further confirmed the importance of treating AD before reaching the advanced stage.

### 2.6 Ab‐TP‐MDNPs Treatment Improves Cognitive Function of AD Mice

**Figure 6:**
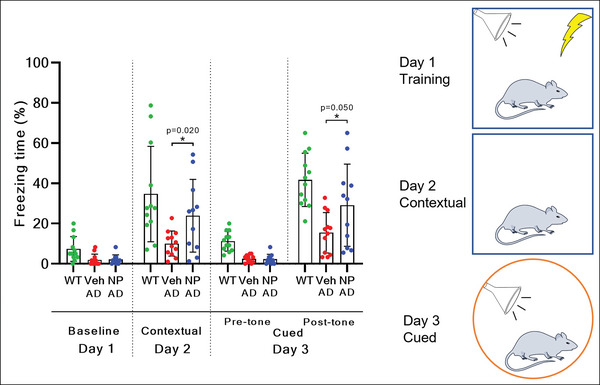
Ab‐TP‐MDNPs improves fear memory. A) Four‐month old AD mice were treated IV with 100 µmol Mn kg^−1^ b.w. of Ab‐TP‐MDNPs or vehicle control once weekly for 8 weeks and fear conditioning test was performed at 6 months of age. Scale bars equal to 100 µm. Data are presented as mean ± SD (*n* = 11–12). Individual values are shown (dots) for Veh and NP treated mice. The asterisk (*) denotes significant difference at *p < 0.05, **p < 0.01, ***p < 0.001, ****p < 0.0001) as compared to vehicle treatment. N.S.—not significant.

Based on our findings that Ab‐TP‐MDNPs treatment remodeled brain microenvironment, we examined the effect of Ab‐TP‐MDNPs on improving the cognitive function (assessed memory) in AD mice. Because AD is a progressive disease in which early intervention is believed to result in better therapeutic outcomes,^[^ ^2^ ^]^ we treated mice at a mild stage of AD (4‐mo age) by weekly injection for 2 months. The effect of Ab‐TP‐MDNPs treatment on cognitive function was then tested at moderate stages of AD (6‐mo age) using fear conditioning tests. Fear conditioning is a classical method to assess associative learning and memory in rodents,^[^ ^29^ ^]^ as such it has been widely employed to assess the cognitive function in TgCRND8 mice and other AD models. Cognitive function in TgCRND8 mice have been shown to normally exhibit significant functional impairment by 6‐mo age.^[^ ^30^, ^31^, ^32^ ^]^ The TgCRND8 mice with vehicle treatment exhibited impairments in both cued and contextual fear indicating the involvement of both hippocampal and amygdala function which is consistent with previous reports.^[^ ^33^ ^]^ Strikingly following the weekly treatment of Ab‐TP‐MDNPs for 2 months, TgCRND8 mice displayed significant improvement in both contextual and cued fear memory scores with an increase in the Freezing Time (%) by 2.38‐fold and 1.89‐fold, respectively, compared to vehicle treated AD littermates (**Figure** **6**).

## 3 Discussion

Our results demonstrate multifunctional effects of Ab‐TP‐MDNPs on remodeling the brain microenvironment of a mouse model of AD through the targeting of several pathological feedback loops in AD. Intravenously injected Ab‐TP‐MDNPs accumulated in disease‐affected neocortical regions, inhibiting several upstream drivers of disease progression, including hypoxia and oxidative stress. These actions ultimately resulted in alteration of key downstream effectors including vascular abnormalities, lymphatic dysfunction and A*β* accumulation.

Mechanistically, Ab‐TP‐MDNPs treatment reduced hypoxia, BACE1, and oxidative stress as a result of a chemical reaction which consumes ROS (H_2_O_2_) and generates local oxygen (Figure 2). Such modification is pertinent given hypoxia and oxidative stress are thought to play a crucial role in AD pathology.^[^ ^4^, ^5^, ^6^, ^7^, ^8^ ^]^ The brain particularly is vulnerable to both hypoxia and oxidative stress due to the long‐lived nature of terminally differentiated neurons, the high lipid content of neurons and support glia such as oligodendrocytes, and the high level of glucose input and oxygen consumption within the brain relative to other organs.^[^ ^8^ ^]^ Following NP treatment, reduced oxidative stress was observed as indicated by reduced oxidation levels in protein and lipid, which could improve the function of neural cells (Figure 2). However, the decrease in HIF‐1*α* levels in AD mouse brains at 24 h post Ab‐TP‐MDNPs treatment was insignificant, probably due to the transient change and short half‐life of HIF‐1*α* that requires an optimal time after the Ab‐TP‐MDNPs treatment for detecting a significant change,^[^ ^7^, ^34^, ^35^, ^36^, ^37^, ^38^ ^]^ while in this study samples were collected at 24 h post last injection to provide sufficient time for Ab‐TP‐MDNPs to be cleared. Consistent with this, Ab‐TP‐MDNPs treatment also reduced observed levels of neuroinflammation, accompanied by a reduction in levels of several inflammatory cytokines with detrimental roles in AD (Figure 3). Examples of these include IL‐1*β* and *α*1‐ACT, responsible for enhancing A*β* load, and IL‐6 promoting tau hyperphosphorylation.^[^ ^39^, ^40^ ^]^

Reduced cerebral blood flow (CBF) in AD can be induced by numerous factors including cerebral amyloid angiopathy, dysfunctional vascular reactivity and perivascular innervation.^[^ ^41^, ^42^, ^43^ ^]^ Cerebral amyloid angiopathy is one of the major causes of vascular dysfunction in AD, as plaque accumulation impinges on blood vessels restricting cerebral blood flow. Still, controversies remain regarding the precise mechanisms and causative role of vascular dysfunction in AD due to its multifaceted nature. Indeed there is evidence to suggest that both pro‐ and antiangiogenic therapies exhibit effects in AD.^[^ ^27^, ^44^, ^45^, ^46^, ^47^ ^]^ Despite this, a preponderance of evidence from both AD and other forms of cognitive impairment suggest that improving vascular tone aids neuronal metabolism and function; given that decreases in blood flow hinders A*β* clearance from the brain inducing hypoxia which further facilitates A*β* production and inflammation.^[^ ^28^, ^48^ ^]^ Thus reductions in both ROS and hypoxia via Ab‐TP‐MDNPs treatment decreases in HIF‐1a and its gene targets BACE1 and VEGF (Figures 2D and 4A) would be expected to improve blood flow through several mechanisms. Analysis of the cerebral blood flow of AD mice by MRI demonstrate that cerebral vascular flow was enhanced following Ab‐TP‐MDNPs treatment. These results, shown in Figure 4, demonstrate that NP treatment resulted in reductions in cerebrovascular plaques and ultimately VEGF and CD31 expression in the brain following Ab‐TP‐MDNP treatment. Thus these treatments both reduced levels of cerebrovascular plaques containing A*β* and improved vascular perfusion and microvessel integrity in AD mice. In other words, Ab‐TP‐MDNPs treatment can inhibit the pathologic feedback loop of reduced blood flow leading to hypoxia, ROS production leading to A*β* accumulation and the progression of AD.

Lymphatic drainage from the brain is considered a major route of A*β* clearance from the brain.^[^ ^16^ ^]^ The effectiveness of Ab‐TP‐MDNPs in this aspect benefited by their selective transport and activity in CSF and cerebrolymphatic regions,^[^ ^23^ ^]^ allowing restoration of lymphatic structures and clearance function. The drainage of Mn and CSF A*β* to the cervical lymph nodes (Figure 5B,C) is indicative of the removal of CSF A*β* through lymph vessels. The significantly increased LYVE‐1 positive lymph vessels (Figure 5D) suggest the normalization of lymphatic structure. Taking this result together with the observed enhancement of vascular perfusion and reduced A*β* plaques (Figure 6A), we can speculate that Ab‐TP‐MDNPs treatment acts to reduce A*β* plaques via these two distinct clearance pathways. Such improved vascular/lymphatic flow likely promotes A*β* and other waste removal while enhancing nutrient exchange required for optimal neural function. With respect to this, we observed an age dependency of the effect of Ab‐TP‐MDNPs treatment on the A*β* plaque reduction. When mice were treated at a moderate stage of the disease, Ab‐TP‐MDNPs were able to reduce A*β* plaques more effectively in the brain than at an advanced stage, where a lesser reduction was detected (Figure S4B, Supporting Information). Nevertheless, the CSF A*β* removed to the cervical lymph nodes by the Ab‐TP‐MDNPs treatment was more profound at the advanced stage than at the moderate stage, whereas the CSF A*β* levels with Veh or NPs treatment (5.6–5.9 ng mL^−1^) were consistently higher than that at the advanced stage without NPs treatment (3.2 ng mL^−1^) (Figure 5C). These results may imply differences in the structural forms of A*β* at different stages of disease progression, with higher levels of oligomeric A*β* potentially cleared by lymphatic drainage mechanisms. Though specific A*β* isoforms cannot be reliably distinguished with the current formulation, we observed that insoluble A*β* plaques are present in greater numbers at advanced stages of disease may potentially be modified through Ab‐TP‐MDNPs treatment to alter plaque solubility and promote clearance. However reductions in A*β* plaques by Ab‐TP‐MDNPs treatment were less effective at advanced stages of disease in addition to greater lymphatic deficiency at advanced stages of disease. Importantly Ab‐TP‐MDNP treatment was observed to improve working memory in this model of AD mice as measured by fear conditioning test (Figure 6B). Interestingly both cued and contextual memory was enhanced by Ab‐TP‐MDNPs treatment, suggesting therapeutic effects of Ab‐TP‐MDNPs across different brain regions. The brain regions involved in contextual memory in AD include the hippocampus, frontal, ventromedial, and cingulate cortex and the amygdala.^[^ ^29^ ^]^ Targeting multiple pathways with Ab‐TP‐MDNPs can thus favorably alter the brain microenvironment inhibiting the feedback loop between pathological signaling involved in disease progression.

Overall, Ab‐TP‐MDNP treatment reduced neuroinflammation, vascular dysfunction, and lymphatic dysfunction, all of which are crucial contributors to AD progression.^[^ ^4^, ^9^, ^49^ ^]^ Improvement in lymphatic A*β* clearance and cerebral vascular flow resulted in the significant removal of A*β* plaques from the AD brain. Additionally, the multifunctional effects of Ab‐TP‐MDNPs treatment improved the fear memory of TgCRND8 mice. Such findings help pave the way for disease‐modifying treatment of AD and encourage further development of effective multitargeted approaches for the treatment of other complex neurodegenerative diseases.

## 4 Experimental Section

### Materials

Soluble corn starch (molecular weight [MW] = 11 000 g mol^−1^), methacrylic acid (MAA), sodium thiosulfate (STS), potassium persulfate (KPS), polysorbate 80 (PS 80), sodium dodecyl sulfate (SDS), *N*‐(3‐(dimethylaminopropyl)‐*N*’‐ethylcarbodiimide hydrochloride (EDC), ethyl arachidate, dextrose, phosphate‐buffered saline (PBS), and Triton X‐100 were purchased from Sigma‐Aldrich, Canada (Oakville, ON, Canada). All chemicals were of analytical grade and used without further purification if not otherwise indicated. Table S1 (Supporting Information) summarizes the materials used for biomarker assays.

### Formulation and Characterization of Ab‐TP‐MDNPs

Brain‐penetrating, A*β*‐targeted, and ROS‐activated nanoparticles (Ab‐TP‐MDNPs) were synthesized as previously described.^[^ ^23^ ^]^ Brain permeable terpolymer of poly(methacrylic) acid (PMMA), polysorbate 80 (PS80), and starch (St) (PMAA‐PS 80‐g‐St) were prepared to formulate polymer‐lipid nanoparticles. Anti‐A*β* antibody 4G8s functionalized terpolymer was obtained by conjugating 4G8 onto the polymers using well‐known 1‐e thyl‐3‐(3‐dimethylaminopropyl)carbodiimide (EDC) chemistry with the concentration of antibody conjugated onto terpolymer brain targeted polymer (TP) was 33 µg 1 mg^−1^ of terpolymer and the Mn content in final nanoparticle is 0.8 mmole Mn g^−1^ Ab‐TP‐MDNPs.^[^ ^23^ ^]^ Briefly, Ab‐TP‐MDNPs were prepared using a modified facile and easily scaled‐up “one‐pot” synthesis method published previously.^[^ ^23^ ^]^ The formulation process starts with the synthesis of precursor MnO_2_ from KMnO_4_ using polyvinyl alcohol as a reducing agent and incorporating these precursor small metal particles into the polymer‐lipid matrix. The final MnO_2_ carrier polymer‐lipid particles were self‐assembled using 4G8 functionalized terpolymers and low melting point lipids under ultrasound sonication at 80% peak (Hielscher UP 100H probe ultrasonicator, Ringwood, NJ). Finally, the obtained nanoparticles solution was purified against doubly distilled deonized water (DDIW) using the Minimate Tangential Flow Filtration Systems (Pall Corporation, Mississauga, ON, CA) and the purified nanoparticles were lyophilized in the presence of a cryoprotectant glucose (1 w/v%) to obtain the dried powder for further use. The particle size and zeta potential of the lyophilized Ab‐TP‐MDNPs were measured using a Malvern Zetasizer Nano ZS (Worcestershire, UK). Detailed nanoparticle morphology was confirmed using transmission electron microscopy (Hitachi H7000) with an accelerating voltage of 75 kV. The concentration of MnO_2_ in the polymer‐lipid formulation was confirmed by measuring the Mn metal ion concentration using an inductively coupled plasma atomic emission spectroscopy (ICP‐AES, Optima 7300 DV ICP‐AES, PerkinElmer Ltd, Boston, MA) method. Based on feed/yield ratio and ICP‐AES results, the quantitative calculation was estimated for the major components of Ab‐TP‐MDNP with 21% of antibody‐terpolymer, 8% of MnO_2_, and 30% of lipids.

### Animals

TgCRND8+ AD transgenic mice (AD mice) and their wild type (WT) littermates were bred at the Krembil Discovery Tower (KDT), Toronto, ON, Canada.^[^ ^50^ ^]^ They were transferred for experimental work to the Spatio‐Temporal Targeting and Amplification of Radiation Response (STTARR) facility within the University Health Network (UHN). All experiments were approved by the Animal Care Committee at UHN. The TgCRND8+ AD mouse strain was developed and characterized as described previously.^[^ ^34^ ^]^ Random distributions of male and female mice were used in the experiments to minimize any possible bias. All animal care procedures undertaken were performed in accordance with the Guide to the Care and Use of Experimental Animals established by the Canadian Council of Animal Care and were approved by the UHN Animal Care Committee (AUP 5703).

### In Vivo Biodistribution and Accumulation of NPs in A*β* Plaque Regions of Mouse Brain

The in vivo biodistribution and CNS accumulation of the NPs were determined using TgCRND8 mice at 6 months of age. HF750‐labeled NPs (100 µmol kg^−1^ animal body weight) were IV injected into the tail vein of mice. At the time points indicated, mice were anesthetized with 2% isoflurane and imaged at *λ*_ex_/_em_ = 754/820 nm using Xenogen IVIS Spectrum Imaging System (PerkinElmer, MA). For the ex vivo imaging of the brains, NPs‐treated mice were euthanized 2 h postinjection, and the brains were dissected and imaged as described above. For ex vivo accumulation of NPs in A*β* plaque regions, mouse brains were collected 2 h post i.v. injection of the Cy5‐labeled NPs, sectioned, and used for further experiments. Briefly, animals were perfused and sacrificed following infusion of PBS, and brains were removed and post‐fixed overnight at 4 °C, followed by cryoprotection in 30% sucrose and sectioning at 20 µm in the coronal plane. Brain section plaques were stained using resorufin,^[^ ^51^ ^]^ and imaged using a Zeiss LSM700 confocal microscope (Carl Zeiss, Jena, Germany) using excitation and emission filters appropriate for detection of the indicated chromophores (Cy3: *λ*_ex_/_em_ = 595/615 nm; Cy5: *λ*_ex_/_em_ = 651/670 nm).

### Inductively Coupled Plasma Atomic Emission Spectroscopy (ICP‐AES)

Elemental analysis of the ICP‐AES samples was performed using the acidic digestion method.^[^ ^23^ ^]^ Briefly, samples were digested in a 2:1 mixture of 70% HNO_3_ and 30% H_2_O_2_ at 85 °C in a hot water bath for 30 min. The digested solutions were diluted (1:5) with distilled water and filtered using 0.22 µm filters (Millex‐GV Syringe Filters, PVDF Durapore Membrane 13 mm diameter, 0.22 µm pore size ethylene oxide sterilized, Millipore, Burlington, MA). The first milliliter of the filtrate was used to saturate the filter, and ≈4 mL of the solution was collected for analysis. The filtrates were analyzed for Mn content using ICP‐AES (Optima7300 DV ICP‐AES, PerkinElmer Ltd, Boston, MA) analysis. The exact tissue weights and dilution volumes were documented for use in final calculations. The concentrations of Mn were measured in triplicate, adjusted to the sample volume and normalized to the brain weight.

### The Enzyme‐Linked Immunosorbent Assay (ELISA)

AD mouse brains were removed from mice 24 h after the last treatment of Ab‐TP‐MDNPs. The whole brain was homogenized in 1 mL of 1X radio‐immunoprecipitation assay buffer (RIPA) buffer (1X, pH 8.0) per 40 mg of brain tissue using a homogenizer (Fisher Scientific High Viscosity Homogenizer PowerGen 1000 S1, Waltham, MA). The homogenate was supplemented with 100 µL of 1X protease inhibitor mixture, and 200 µL each of 1X phosphatase inhibitor mixtures 1 and 2 (Sigma‐Aldrich, St. Louis, MO) per 10 mL of ice‐cold buffer and centrifuged at 14 000 × g for 10 min. The supernatant was then collected and used to measure protein content by ELISA as per the manufacturer's protocols (Bio‐Rad xMark Microplate Absorbance Spectrophotometer, Hercules, CA).

### Dihydroethidium Assay

Intracellular ROS levels were measured as follows.^[^ ^52^ ^]^ Briefly, dissected brains were homogenized using a homogenizer (Fisher Scientific High Viscosity Homogenizer PowerGen 1000 S1, Waltham, MA) in 0.01 m PBS (pH 7.2–7.4). To the homogenized tissue suspension (0.4 mg mL^−1^), the cell‐permeable probe dihydroethidium (DHE, 5 µm) (Sigma‐Aldrich, St. Louis, MO) was added and incubated for 30 min at 37 °C in the dark. The fluorescence intensity of the suspension was measured using a plate reader (BioTek Synergy Neo2 Hybrid Multi‐Mode Reader, Winooski, VT) at 530 nm as the excitation and 630 nm as the emission wavelength. The normalized data were expressed as a value relative to 100%.

### Immunohistochemistry

Mouse brain 20 µm sagittal cryo‐sections were first treated with 3% H_2_O_2_ for 15 min at room temperature to inactivate endogenous peroxidases (Leica CM1950, Wetzlar, Germany). Sections were stained with primary antibodies against CD31 (1:50, overnight) or LYVE‐1 (1:100, 1 h). Alternatively, mouse brains were harvested after perfusion with saline and fixed with 10% buffered formalin and embedded in paraffin. Sections were stained with primary antibodies against CA9, CD3, IBA‐1, GFAP, or 4G8. After washing steps, the secondary antibody reagents were added following the manufacturer's protocol. Slides were visualized under a fluorescence whole slide scanning microscope (Olympus Upright BX50 microscope, Tokyo, Japan) or Zeiss Mirax Slide Scanner at 20X magnification with a Zeiss 20X/0.8 objective lens (Carl Zeiss AG, Jena, TH, Germany) and a Marlin F146‐C CCD camera (Sony, Tokyo, Japan) at 20X magnification. Images were analyzed and quantified using ImageJ software based on the percentage of positive stain per area. Suppliers of the antibodies are described in Table S1 (Supporting Information).

### Plaque Staining and Quantification

TgCRND8 brains were prepared for immunostaining as described previously.^[^ ^53^ ^]^ For total plaque staining, every fifth section of 25 paraffin‐embedded sections was stained with the 4G8 antibody to A*β* and detected with 3,3'‐diaminobenzidine (DAB). Slides were scanned using the Mirax Scan version 1.11 software and Zeiss Mirax Slide Scanner at 20X magnification with a Zeiss 20X/0.8 objective lens (Carl Zeiss AG, Jena, TH, Germany) and a Marlin F146‐C CCD camera (Sony, Tokyo, Japan), operated at room temperature. The rendered digital images were analyzed using a color deconvolution algorithm. Red–green–blue values were determined for both the applied hematoxylin and DAB stains. Diaminobenzadine was chosen as the positive color channel for identifying and quantifying A*β*‐stained plaques within different areas of the brain (cortex and hippocampus). Furthermore, recognition and measurement of dense and diffuse plaque‐stained areas were achieved by setting the threshold values of color intensity. The strong positive threshold was set to 140, correlating with dense staining. The medium positive threshold was set to 175, correlating with medium/diffuse staining, and the weak positive threshold was set to 0. In this way, the amyloid‐positive area, as well as intensity of A*β* staining, was quantified in different brain regions, allowing for the quick, objective comparison between brains from different animals.

For cerebrovascular plaque staining, sections were prepared as described previously.^[^ ^51^ ^]^ Sections were dewaxed and blocked in PBS containing 0.1% Triton X‐100, 0.2% casein with 1% BSA at room temperature for 45 min. Sections were then permeabilized in 0.25% Triton X‐100 (in PBS) for 30 min at room temperature and then incubated in 1 µm resorufin for 30 min at room temperature in 0.25% Triton X‐100 in PBS. After this, sections were washed three times in PBS, once in 50% ethanol (EtOH) in PBS, followed by three additional PBS washes. Sections were cover‐slipped and slides were visualized under a fluorescence whole slide scanning microscope (Olympus Upright BX50 microscope, Tokyo, Japan) or light microscopy at 20X magnification. All visible cortical vessels were imaged, and all images used for quantification were obtained with an exposure time of 1 s. Cross‐sectional intensity was quantified using the NIH ImageJ software.

### Magnetic Resonance Imaging Assessment of Cerebral Blood Flow and BBB Permeability

In vivo MR images of mouse brains were acquired using a 7 Tesla micro‐MRI (BioSpec 70/30 USR, Bruker, Ettlingen, BW, Germany) equipped with the B‐GA12 gradient coil, 7.2 cm inner diameter linearly polarized cylindrical volume coil for radiofrequency (RF) transmission, and a dedicated murine brain receive‐only RF coil for MR signal reception. Mice were anesthetized using 1.8% isoflurane and imaged in a prone position on a custom slider bed. A pneumatic pillow‐fixed above the thorax/abdomen provided a signal for respiratory monitoring (SA Instruments, Stony Brook, NY). Mice were also prepared via tail vein cannulation with a 27 G needle and a precision line to enable automated contrast agent injection during imaging (Harvard Apparatus, Holliston, MA).

A single vertical section was identified through the mid‐brain for quantitative imaging. The CBF was assessed using FAIR (flow‐sensitive alternating inversion recovery) technique incorporating a centrically‐ordered steady‐state free precession (FISP) readout.^[^ ^54^ ^]^ FAIR‐FISP isolates perfusion, as an elevated T1‐weighted signal following slice‐selection were compared to nonselective inversion preparations, as per the following equation: CBF = Λ/2TI * (SS(TI)– NS(TI)/M0 * exp(TI/T1) (mL/(100 g min^−1^), where “SS” and “NS” denote slice‐selective and nonselective measurements, M0 is the reference FISP signal, and Λ is the blood–brain partition coefficient, defined as the ratio between water concentration per g brain tissue and per mL blood. The T1 longitudinal relaxation time was measured from the variable‐TR approach noted below, and TI is the inversion time (1400 ms). The FAIR optimization utilized 4.6 ms s adiabatic RF pulses for NS and SS inversion (≈3826 Hz bandwidth; 3 mm slice package margin around SS inversion pulse), with 12 s allotted for T1 recovery prior to RF inversion. FISP readout parameters included 20 dummy echoes, TE/TR of 1.27/2.55 ms, 60° flip angle, 80 × 80 matrix over 18 × 18 mm field‐of‐view, for 225 × 225 µm in‐plane resolution, 1.5 mm slice thickness, 16 averages, and 3 min 38 s for NS and SS acquisitions.

Endogenous T1 mapping was acquired as an input parameter for CBF calculation, and for BBB permeability assessment via T1 mapping reacquisition prior to and following intravenous injection of Gadovist at 1.2 mmol kg^−1^. T1 mapping used a variable‐TR spin‐echo Rapid Acquisition with Relaxation Enhancement (RARE) technique (7 TR values of 250, 500, 750, 1000, 1500, 2500, 4000 ms; RARE factor 2; 80 × 80 matrix over 18×18 mm field‐of‐view, for 225×225 µm in‐plane resolution, 1.5 mm slice thickness; 1 average; 5 min 15 sec).

T1 MRI maps in Dicom format were generated via in‐line processing on the Bruker console. R1 (1/T1) and R1 difference (1/T1, post — 1/T1, pre) maps were generated in Matlab (The Mathworks, Natick, MA). The R1 difference is linear with Gd‐DTPA concentration, enabling approximation of parenchymal Gd‐DTPA concentration as a BBB permeability biomarker on a per voxel basis, assuming T1 relaxivity of 4.5 L mmol^−1^ s^−1^.^[^ ^55^ ^]^ Dicom CBF maps were generated using custom Matlab scripts. Quantitative measurements in individual images were performed in registered manually segmented brain cortical and subcortical volumes using MIPAV software (National Institutes of Health, Bethesda, MD).

### Fear Conditioning Test

Mice were tested for cued and contextual fear memory as previously described.^[^ ^31^, ^32^ ^]^ Briefly, mice were trained and tested in operant chambers on 3 consecutive days in the cued and contextual fear conditioning paradigm. On Day 1, mice were placed into Context A (grid floor, isopropyl alcohol scent, houselights at 100%) and allowed to explore for 120 s (baseline) prior to three 30 s tone/shock pairings (30 s, 4 kHz pure tone coterminating with a 2 s scrambled 0.6 mA foot‐shock). Each tone/shock pairing was separated by 30 s of exploration time and animals were given 30 s to explore following the final tone/shock pairing (300 s total). On Day 2, mice were replaced into Context A and allowed to explore for 180 s without the tone. Day 3, mice were placed into Context B (black plastic wall and floor, lemon scent, houselights at 50%) and allowed to explore for 180 s in the constant presence of the 4 kHz pure tone. Freezing was defined as a lack of movement except that required for respiration. Memory for the context (contextual memory) or the tone (cued memory) for each animal was obtained by subtracting the percent freezing during baseline from the percent freezing on Day 2 or Day 3, respectively. Freezing behavior was recorded remotely and analyzed using Freeze Monitor System software (San Diego Instruments, USA).

### In Vitro Study of Ab‐TP‐MDNPs in Reducing Oxidative Stress‐Induced Damage to Neurons

Primary cortical neurons were isolated from the cerebral cortex on embryonic day 15 (E15.5.) CD‐1 mice as previously described.^[^ ^56^ ^]^ Eight well plates were precoated with laminin (Sigma‐Aldrich, USA) and poly‐*D*‐lysine (Cultrex Poly‐*D*‐Lysine, Amsbio, USA) (1:20) for 1 h at 37 °C. CNS cortical neurons were then dissociated in *α*MEM medium (Gibco, ThermoFisher Scientific, CA) within 15 min of sacrifice and cells were plated in the neurobasal medium plus 2% B‐27 serum‐free supplement (Gibco, ThermoFisher Scientific, USA), 1% penicillin‐streptomycin‐glutamine (Gibco, ThermoFisher Scientific, USA), 10% fetal bovine serum at a concentration of 60 000 or 120 000 cells mm^−2^. Following culture for seven days at 37 °C, 5% CO_2_, cells were treated with 15 µm Ab‐TP‐MDNP, 50 µm H_2_O_2_, and a combination of 15 µm Ab‐TP‐MDNP/50 µm H_2_O_2_ and incubated for 6 h at 37 °C, 5% CO_2_. At 5.75 h, 10 µL of 0.5 µg mL^−1^ Hoechst 33 342 and 2 µL of 50 µm calcein AM (Cedarlane, ON, Canada) solution was added to the cells and incubated for 15 min. Cells were then washed three times with fresh media and replaced with 200 µL of fresh medium. Finally, neuronal morphology was imaged by using a confocal laser scanning microscope (Zeiss LSM 700 confocal microscope, Germany) with the filter for FITC for Calcein AM: *λ*_ex_/_em_ = 490/525 nm.

### Statistical Analysis

Statistical analysis was performed using GraphPad Prism 9 (San Diego, CA). Data were analyzed using either *t*‐test, Fisher's exact test, or ANOVA test followed by Tukey's multiple comparison test as appropriate. Data are represented as mean ± SD. *p* < 0.05 was considered statistically significant.

## Conflict of Interest

The authors declare no conflict of interest.

## Author Contributions

Conceptualization: X.Y.W., C.H., J.T.H., P.E.F., E.P. Methodology: E.P., W.F., C.H., J.T.H., P.E.F., M.Z., R.P.B., X.Y.W. Investigation: E.P., L.Y.L., C.H., A.A., T.A., W.F., R.O., J.T.H. Visualization: E.P., T.A., X.Y.W. Funding acquisition: X.Y.W., J.T.H., P.E.F. Project administration: X.Y.W., C.H. Supervision: X.Y.W., J.T.H., P.E.F. Writing—original draft: E.P. Writing—review & editing: E.P., L.Y.L., C.H., A.A., T.A., W.F., A.M.R., J.T.H., P.E.F., X.Y.W.

## Supporting information

Supporting Information

## Data Availability

The data that support the findings of this study are available from the corresponding author upon reasonable request.
