# Supplementary material for: Brain‐Penetrating and Disease Site‐Targeting Manganese Dioxide‐Polymer‐Lipid Hybrid Nanoparticles Remodel Microenvironment of Alzheimer's Disease by Regulating Multiple Pathological Pathways
Source: Adv Sci (Weinh). 2023 Feb 19;10(12):2207238. doi: 10.1002/advs.202207238 (PMC10131868; doi:10.1002/advs.202207238)
Supplement: Supplementary file 1 — Supporting Information [file ADVS-10-2207238-s001.pdf]

ADVANCED SCIENCE
Open Access

Supporting Information

for *Adv. Sci.*, DOI 10.1002/advs.202207238

Brain-Penetrating and Disease Site-Targeting Manganese Dioxide-Polymer-Lipid Hybrid Nanoparticles Remodel Microenvironment of Alzheimer's Disease by Regulating Multiple Pathological Pathways

*Elliya Park, Lily Yi Li, Chunsheng He, Azhar Z. Abbasi, Taksim Ahmed, Warren D. Foltz, Regan O'Flaherty, Maham Zain, Robert P. Bonin, Andrew M. Rauth, Paul E. Fraser, Jeffrey T. Henderson and Xiao Yu Wu**

Supporting Information

Brain-penetrating Disease Site-targeting Manganese Dioxide-polymer-lipid Hybrid Nanoparticles Remodel Microenvironment of Alzheimer's Disease by Treating Multiple Pathological Pathways

*Elliya Park, Lily Yi Li, Chunsheng He, Azhar Z. Abbasi, Taksim Ahmed, Warren Foltz, Regan O'Flaherty, Maham Zain, Robert P. Bonin, Andrew M. Rauth, Paul E. Fraser, Jeffrey T. Henderson, and Xiao Yu Wu**

E-mail: sxy.wu@utoronto.ca

E. Park, L. Y. Li, C. He, A. Z. Abbasi, T. Ahmed, M, Zain, R. P. Bonin, J. T. Henderson, X. Y. Wu
144 College St, Leslie Dan Faculty of Pharmacy, University of Toronto, Toronto, ON, M5S 3M2, Canada

W. D. Foltz
149 College St, Department of Radiation Oncology, University Health Network, Toronto, ON, M5T 1P5, Canada

A. M. Rauth
101 College St, Departments of Medical Biophysics and Radiation Oncology, University of Toronto, Toronto, ON, M5G 1L7, Canada

P. E. Fraser, R. O'Flaherty
135 Nassau St, Tanz Centre for Research in Neurodegenerative Diseases, Department of Medical Biophysics, University of Toronto, Toronto, ON, M5T 1M8, Canada

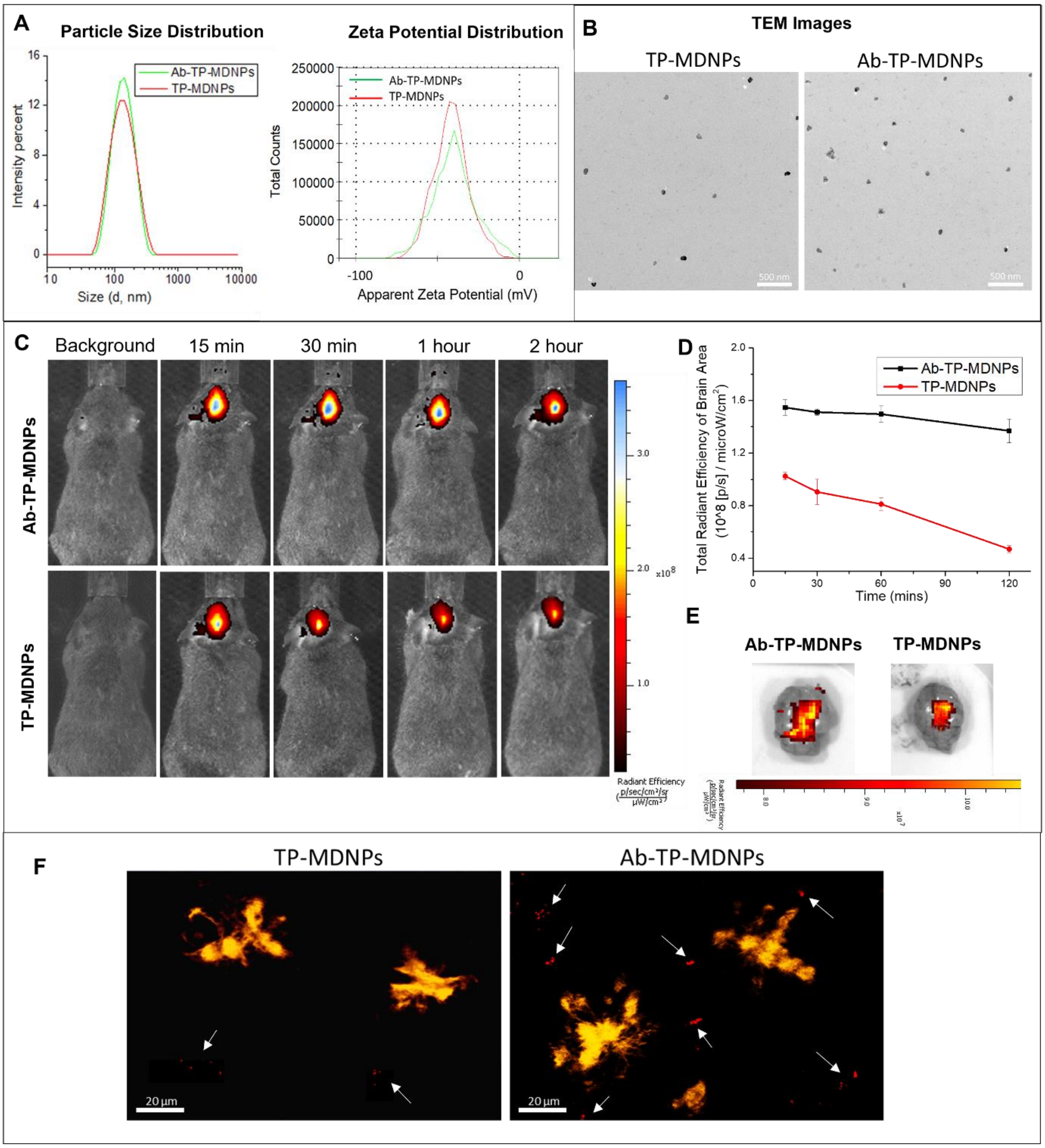

Figure S1. Characterization of TP-MDNPs and Ab-TP-MDNPs. A) Distribution of particle size and zeta potential of TP-MDNPs and Ab-TP-MDNPs determined using a Zetasizer (d.nm = diameter in nanometer), B) TEM images of Ab-TP-MDNPs and TP-MDNPs. Scale bars equal 100 nm. C) Representative *in vivo* images of AD mouse following IV injection of fluorescence labelled Ab-TP-MDNPs over 120 mins. D) Quantitative plots of fluorescence intensity profiles of near-infrared dye-labeled NPs in the brain region of AD mice up to 120 mins (n=3 per group). E) Representative *ex vivo* brain images of fluorescent intensity acquired at 120 min post-administration. F) CLSM images of brain sections from AD mouse at 120 min post-administration. Aβ plaques are shown as orange and Cy5-TP-MDNPs or Cy5-Ab-TP-MDNPs in red. Arrows indicate nanoparticles.

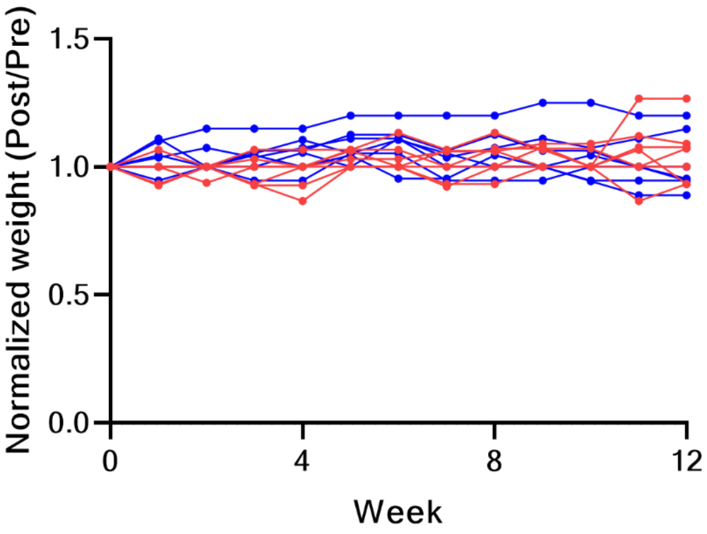

Figure S2. Bodyweight changes after long-term Ab-TP-MDNPs treatment. To observe the safety of long-term Ab-TP-MDNPs treatment, 100 µmol/kg b.w. Ab-TP-MDNPs were administered once weekly to 4 mo-old TgCRND9 mice (random distribution of male and female mice) and the bodyweight is measured for 12 weeks. The body weight of each mouse was normalized by their initial weight per individual mouse (n = 7 per group). Red dots are for Veh-treated mice and blue dots are for NP-treated mice. There was no significant difference between the treatment groups. Similarly, 2 weeks of Ab-TP-MDNPs treatment twice weekly used in the study had no significant difference in their body weight (n=6-7 per group)

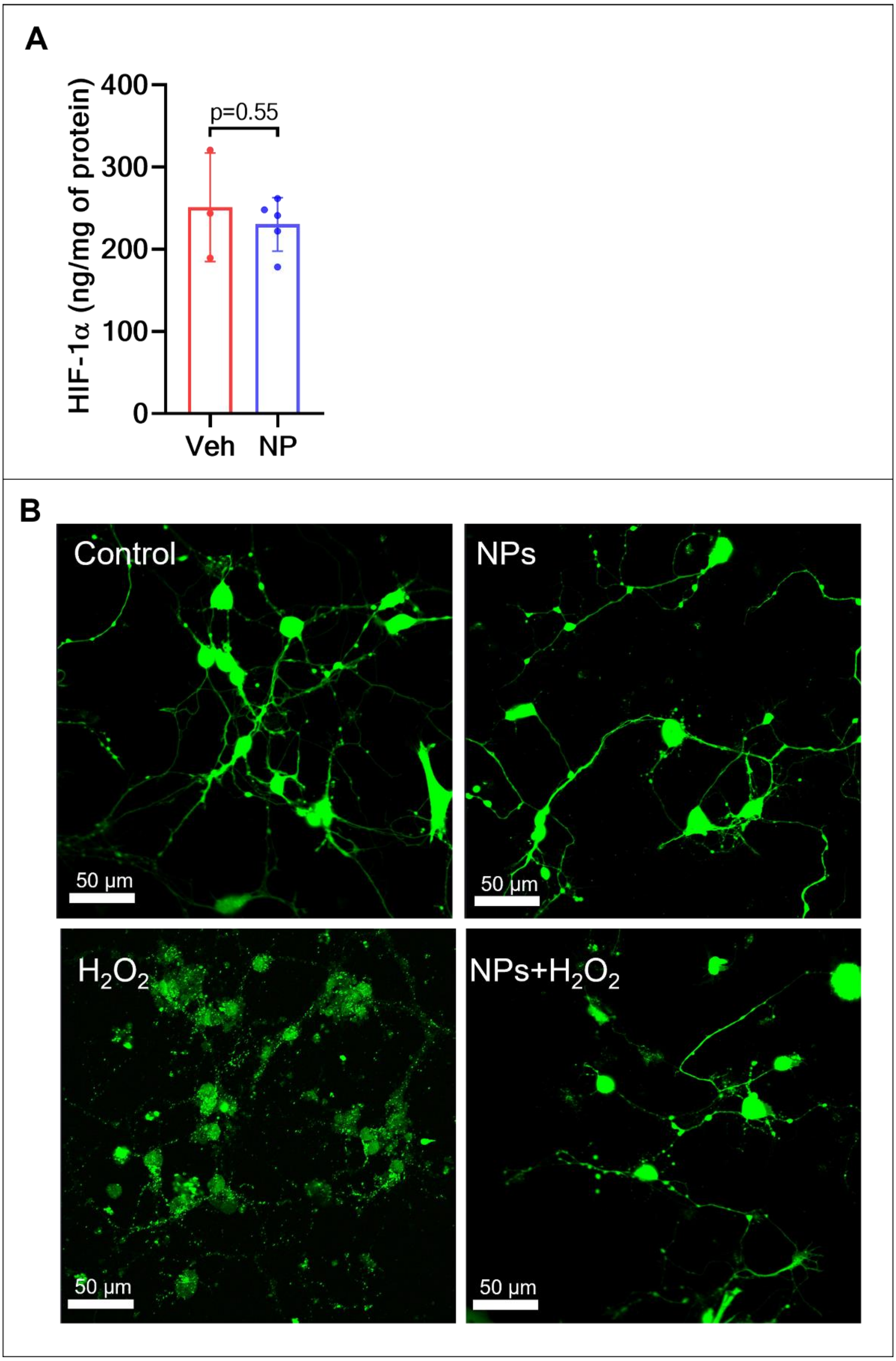

Figure S3. Ab-TP-MDNPs reduces oxidative stress-induced dendritic damages but not HIF-1α. A) Six-mo old AD mice (random distribution, male and female mice) were treated IV with 100 μmol Mn/kg body weight (b.w.) of Ab-TP-MDNPs (NP) or Veh control twice weekly for 2 weeks and measurements were made on day 15. Levels of HIF-1α as measured by ELISA in AD brain homogenates. The data are presented as mean ± SD (n = 3-5 per group). Individual values are shown

(dots) for vehicle and NP treated mice. Asterisk(s) (*) denotes a significant difference at *p<0.05, **p<0.01) compared to Veh treatment. N.S. – not significant. B) CLSM images of neuronal morphology *in vitro* of mouse cortical neurons with or without Ab-TP-MDNPs treatment after exposure to 50 µM H_2O_2. Scale bars equal 50 µm.

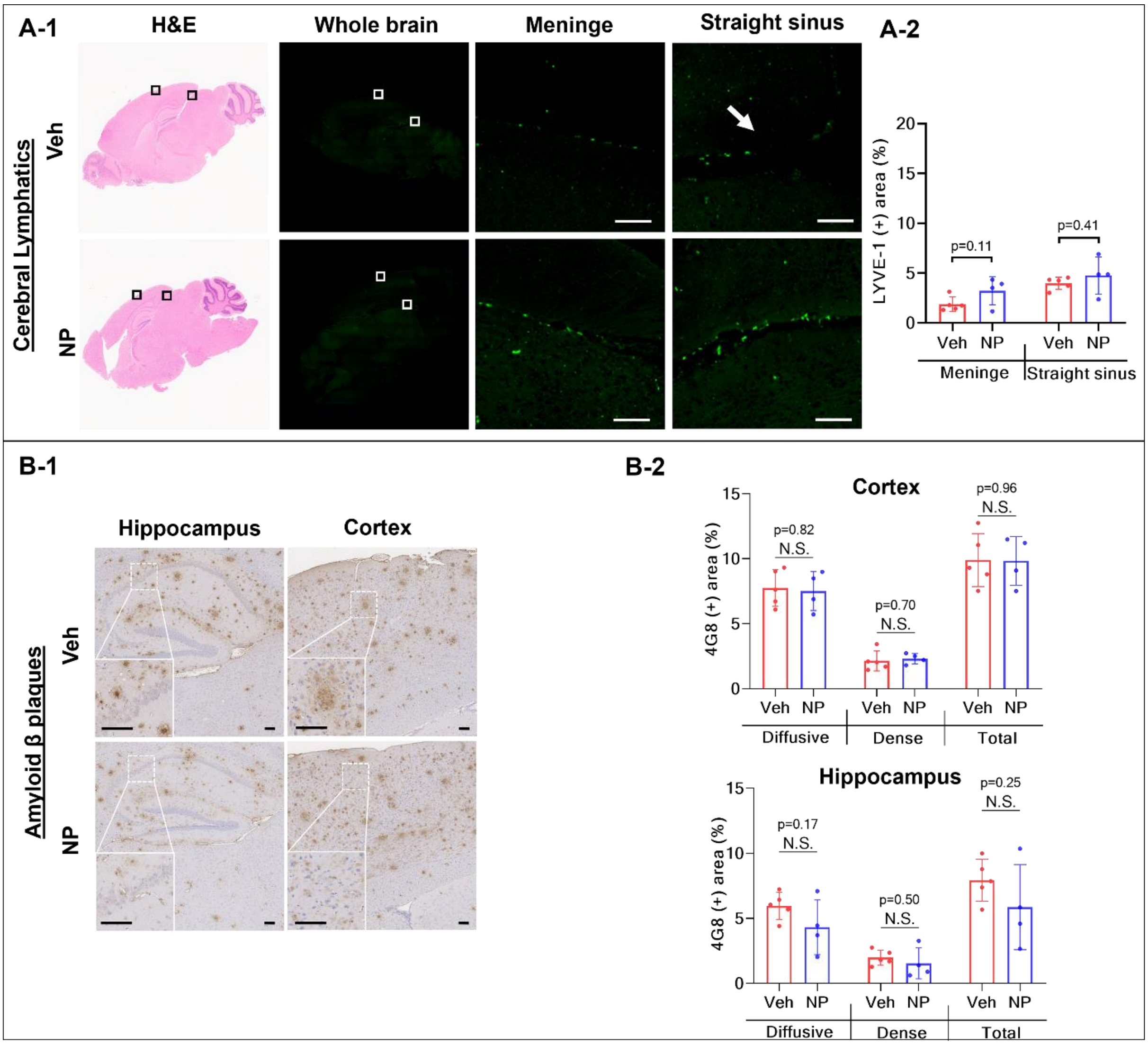

Figure S4. Nine-mo-old AD mice (random distribution of male and female mice) were IV treated with 100 µmol Mn/kg b.w. of Ab-TP-MDNPs or Veh twice weekly for 2 weeks. A) Hematoxylin/eosin overview with enlargements demonstrating LYVE-1 staining for cerebral lymphatics in the meninge and straight sinus of AD mouse brains (A-1). Scale bars equal 100 µm, results quantified in (A-2). Scale bars equal to 100 µm. B) Immunohistochemistry of 4G8 at an advanced stage of AD mice (9-moold). Images of immunohistochemistry staining of 4G8 for Aβ (B1) and quantitative plots of 4G8-positive area (B2) in the hippocampus and cortex of 9-mo AD mice brains. Individual mouse values are shown by red dots for Veh treated mice and blue dots for NP treated mice. The data are presented as mean ± SD (n=4-5 per group). The asterisk (*) denotes a significant difference (N.S. (not significant) p>0.05, *p< 0.05) as compared to Veh treatment.

Pathway	Purpose	Biomarker	Method	Supplier (Catalog number)
**Oxidative stress, hypoxia**	Hypoxia	Carbonic anhydrase 9	IHC, ELISA	Novus biologicals (nb100-417, NBP2-71283)
	Total ROS	Dihydroethidium	DHE assay	Sigma-Alderich (D7008)
	Protein oxidation	Protein carbonyl	DNPH-carbonyl assay	Abcam (ab126287)
	Lipid peroxidation	8-Isoprostane	ELISA	Abcam (ab175819)
	DNA oxidation	8-OHdG	ELISA	Abcam (ab201734)
	Hypoxia downstream	BACE1	ELISA	Mybiosource (MBS2511578)
**Inflammation**	Reactive microglia	Iba-1	IHC	Novus biologicals (nb100-1028)
	Reactive astrocytes	GFAP	IHC	Biolegend (801103)
	Infiltrated CD3+ cell	CD3	IHC	Abcam (ab11089)
	Inflammatory cytokines	IL-1β	IHC, ELISA	Abcam (ab9722, ab100704)
		IL-6	ELISA	Invitrogen (BMS603-2)
		TNF-α	ELISA	Invitrogen (BMS607-3)
		α1-ACT	ELISA	Mybiosource (MBS2064807)
**Vascular function**	Vascular flow	FAIR-FISP	MRI	
	BBB permeability	Gd permeability	MRI	Bayer AG (Gadavist)
	Vascular structure	CD31	IF	BD Biosciences (550274)
	Vascular cytokine	VEGF	ELISA	Novus Biologicals (NBP1-92679)
**Aβ clearance**	Lymphatic structure	LYVE-1	IF	Novus biologicals (NB6001008)
	Biodistribution in cerebral lymphatics	Mn in cervical lymph nodes after NP inj.	ICP-AES	

	Aβ clearance	CSF Aβ	ELISA	Thermofisher (KHB3544)
	Aβ plaques	4G8	IHC	Biolegend (800703)
	Vascular Aβ	Resorufin	IF	Sigma-Alderich (73144)

Table S1. Supplementary List of Pathways – Materials Used. The experimental methods used in the study are summarized in Table S1. The effects of Ab-TP-MDNPs treatment in 4 different pathways have been investigated in the TgCRND8 model.
